# Creating human locus coeruleus organoids to study the role of the locus coeruleus in Alzheimer’s disease

**DOI:** 10.1002/alz.089378

**Published:** 2025-01-03

**Authors:** Margaret M Tish, Fikri Birey, David Weinshenker, Zihui Ou

**Affiliations:** ^1^ Emory University School of Medicine, Atlanta, GA USA; ^2^ Emory University, Atlanta, GA USA

## Abstract

**Background:**

The locus coeruleus (LC), is the first brain region to develop hyperphosphorylated tau (ptau) inclusions in Alzheimer’s disease (AD) and undergoes catastrophic degeneration in later stages of the disease. Importantly, the LC is the main noradrenergic nucleus in the brain and source of NE in the forebrain, and dysregulation of the neurotransmitter norepinephrine (NE) is associated with AD symptoms, as its release in the forebrain regulates attention, arousal, stress response, and learning and memory. Moreover, the LC may transmit pathogenic tau to the forebrain via its extensive projections. However, nearly all information about the LC in AD has been derived from rodent models and postmortem or human imaging studies. It is not known how the early ptau accumulation in the human LC affects neuronal function, NE transmission, or pathology propagation. We created organoids, which are 3D human cell culture models derived from human induced pluripotent stem cells, that possess many aspects of human LC structure.

**Method:**

Organoids containing neurons resembling the LC were generated from human induced pluripotent stem cells using growth factors that induce a hindbrain fate. These organoids were then characterized using HPLC, immunohistochemistry, RNA‐scope and RNA‐sequencing.

**Result:**

LC organoids contained higher levels of norepinephrine and dopamine than cortical organoids. They also expressed many of the proteins and RNA that are indicative of and enriched LC‐NE neurons, including tyrosine hydroxylase, dopamine beta‐hydroxylase, the NE transporter, Phox2A, and Phox2B. Additionally, bulk RNA‐seq revealed that compared to cortical organoids, the LC organoid transcriptome more closely resembled the human LC.

**Conclusion:**

LC organoids are distinct from cortical organoids and contain many markers of human LC neurons. Studying the structure and function of LC organoids derived from both healthy controls and AD patients may provide new insights into the role of the LC in AD.

